# Concerted regulation of renal plasma flow and glomerular filtration rate by renal dopamine and NOS I in rats on high salt intake

**DOI:** 10.14814/phy2.13202

**Published:** 2017-03-29

**Authors:** Mariano E. Ibarra, Maria F. Albertoni Borghese, Mónica P. Majowicz, María C. Ortiz, Fabián Loidl, Manuel Rey‐Funes, Luis A. Di Ciano, Fernando R. Ibarra

**Affiliations:** ^1^ Laboratorio de Neuropatología ExperimentalInstituto de Biología Celular y Neurociencia “Prof. E. De Robertis” (IBCN)Facultad de MedicinaUniversidad de Buenos AiresBuenos AiresArgentina; ^2^ Cátedra de Biología Celular y MolecularFacultad de Farmacia y BioquímicaUniversidad de Buenos AiresBuenos AiresArgentina; ^3^ Laboratorio de RiñónInstituto de Investigaciones Médicas A. LanariUniversidad de Buenos AiresBuenos AiresArgentina; ^4^ Departamento de Ciencias FisiológicasFacultad de MedicinaUniversidad de Buenos AiresBuenos AiresArgentina

**Keywords:** High salt intake, NOS I in MD, Regulation of Renal Plasma Flow, Renal Dopamine

## Abstract

Under high sodium intake renal dopamine (DA) increases while NOS I expression in macula densa cells (MD) decreases. To explore whether renal DA and NOS I, linked to natriuresis and to the stability of the tubuloglomerular feedback, respectively, act in concert to regulate renal plasma flow (RPF) and glomerular filtration rate (GFR). Male Wistar rats were studied under a normal sodium intake (NS, NaCl 0.24%) or a high sodium intake (HS, NaCl 1% in drinking water) during the 5 days of the study. For the last two days, the specific D_1_‐like receptor antagonist SCH 23390 (1 mg kg bwt^−1^ day^−1^, sc) or a vehicle was administered. HS intake increased natriuresis, diuresis, and urinary DA while it decreased cortical NOS I expression (*P* < 0.05 vs. NS), Nicotinamide adenine dinucleotide phosphate diaphorase (NADPH‐d) activity in MD (*P* < 0.001 vs. NS) and cortical nitrates+nitrites (NOx) production (NS 2.04 ± 0.22 vs. HS 1.28 ± 0.10 nmol mg protein^−1^, *P* < 0.01). Treatment with SCH 23390 to rats on HS sharply decreased hydroelectrolyte excretion (*P* < 0.001 vs. HS) while NOS I expression, NADPH‐d activity and NOx production increased (*P* < 0.05 vs. HS for NOS I and *P* < 0.001 vs. HS for NADPH‐d and NOx). SCH 23390 increased RPF and GFR in HS rats (*P* < 0.01 HS+SCH vs. HS). It did not cause variations in NS rats. Results indicate that when NS intake is shifted to a prolonged high sodium intake, renal DA through the D_1_R, and NOS I in MD cells act in concert to regulate RPF and GFR to stabilize the delivery of NaCl to the distal nephron.

## Introduction

Renal perfusion, measured as renal plasma flow (RPF) or glomerular filtration rate (GFR), is strictly regulated within a range of mean arterial pressure (MAP) levels to avoid serious distortions in renal function secondary to changes in MAP (Selkurt et al. 1949; Carlström et al. 2015). This adjustment of renal perfusion under varying conditions is referred to as an autoregulation phenomenon. One of the mechanisms involved in the autoregulation of RPF is the tubuloglomerular feedback (TGF) which induces a constriction of the glomerular afferent arteriole (AA) when a high concentration of NaCl reaches macula densa (MD) cells (Moore et al. 1979; Schnermann 2003). The excessive delivery of NaCl to distal portions of the nephron can be the consequence of an increase in GFR secondary to an increment in arterial pressure or reduction in proximal tubules′ fluid reabsorption (Palmer & Schnermann 2015). It can also be due to an increase in salt intake which causes higher concentrations of NaCl reaching MD cells. Classic micropuncture studies show that TGF mechanism is developed in frame‐times between seconds and few minutes (Schnermann 2003); but TGF regulates RPF under more chronic and stable conditions, like high sodium intake as well (Gao et al. 2011). The AA constriction reduces Na^+^ filtered load and prevents a great amount of sodium from being delivered to distal portions of the nephron. Actual evidence suggests that AA constriction is caused by adenosine or other purinergic mediators released by MD cells (Just and Arendshorst 2007).

MD cells are endowed with specific ionic transporters at the luminal and basolateral membranes and participate in the paracrine regulation of neighboring cell activity (Bell and Lapointe 1997; Persson and Bachmann 2000). Cells of MD express the neuronal isoform of nitric oxide synthase (NOS I), which is also expressed in other renal structures. However, MD cells are the site of its highest expression in the renal cortex (Tojo et al. 1994). NOS I activity in MD produces nitric oxide (NO). NO released by MD blunts the AA constriction produced in response to TGF and to the vascular myogenic mechanism (Imig and Roman 1992; Arima and Ito 2000; Just and Arendshorst 2005). In addition, it has been shown that NOS I expression in renal cortex and in MD cells is inversely related to sodium intake and thus rats on a high sodium diet have less NOS I mRNA and less NOS I protein expression than control rats (Singh et al. 1996; Tojo et al. 2000).

Renal dopamine (DA) is synthesized in the renal epithelial cells of proximal tubules (PT). DA in the kidney is the result of the decarboxylation of l‐dopa, which enters to proximal cells once ultrafiltered from plasma (Aperia 2000). Renal DA is continuously produced by PT (Ibarra et al. 2005), but its production is modulated by both changes in Na^+^ intake and in extracellular fluid volume. High sodium intake and extracellular fluid volume expansion induce an increase in the amount and activity of urinary DA (Hansell and Fasching 1991; Lee 1993; Wang et al. 1997; Reddy et al. 1998) while low sodium intake and extracellular volume contraction decrease the daily amount of urinary DA (De Luca Sarobe et al. 2005). Besides, diuresis and natriuresis are clearly impaired during a HS intake or volume expansion when either endogenous DA production is inhibited or D_1_‐like receptors are blocked (Chen and Lokhandwala 1992; De Luca Sarobe et al. 2010; Di Ciano et al. 2015).

Then, since in HS intake the synthesis and effects of renal DA are increased and, on the other hand, it has been reported that NO released by MD is diminished to allow a full expression of the TGF mechanism, these facts prompted us to investigate whether DA produced by proximal tubule cells and NO released by MD NOS I act in concert to regulate RPF and GFR under the condition of a high sodium intake.

## Material and Methods

### Experimental design

Male Wistar rats from the Animal Breeding Facility of Instituto de Investigaciones Médicas A. Lanari, University of Buenos Aires, with a body weight of 250–300 g were used for this study. All protocols were performed according to the guidelines recommended by the European Convention for the Protection of Vertebrate Animals used for Experimental and other Scientific Purposes and were reviewed by the Local Institutional Committee for Animal Welfare of the Instituto de Investigaciones Médicas A. Lanari, Universidad de Buenos Aires (CICUAL). They were housed at 22.0 ± 2.2°C with a 12:12 h dark/light cycle.

Rats were assigned into groups according to normal or high sodium intake for 5 days. In order to minimize the effect of stress all rats were previously acclimatized to individual cages.

Then, experimental treatments were performed as follows:

1. Normal salt group (NS): standard diet (Cooperación, San Nicolás, Argentina) (0.24% NaCl) and tap water ad libitum.
2. High salt group (HS): rats received 1% NaCl in drinking water and standard diet.

Two days before clearance studies subgroups of NS or HS rats were treated with the D_1_‐like receptor antagonist SCH 23390 (R (+)‐SCH‐23390 hydrochloride, Sigma‐Aldrich, St. Louis, MO) (1 mg kg bwt^−1^ s.c. twice a day, dissolved in 100 *μ*L of normal saline) or with vehicle (100 *μ*L of normal saline s.c.), resulting in a total of four groups: NS, HS, NS+SCH (normal salt, treated with SCH 23390), and HS+SCH (high salt, treated with SCH 23390).

All the animals were placed in metabolic cages during the 5 days of the study to allow for 24 h urine collections. For DA analysis 24‐h urine samples were collected on 500 *μ*L 6N HCl to prevent DA degradation. Water excretion and urine electrolyte composition were also measured in these samples. Systolic blood pressure (SBP) was recorded by an indirect technique (tail cuff method) which involves the occlusion of circulation in the tail with an annular cuff and the detection of pulse as the cuff pressure is lowered using a Physiograph MK III S (Narco Biosystem, Austin, TX). The SBP considered resulted from a mean of 10 recordings obtained each time.

After the five‐day collection, animals were anesthetized with Inactin (50 mg kg bwt^−1^, i.p.) and surgically prepared for a renal clearance experiment as described previously (Ibarra et al. 1998). The trachea was cannulated to facilitate breathing. Catheters were placed in the carotid artery for blood sampling and jugular vein for i.v. infusions. Enough inulin (Inutest, Linz, Austria) and paraaminohippurate (Merck, Sharp & Dohme, West Point, PA) to provide plasmatic concentrations of 0.2 and 0.02 mg mL^−1^, respectively, were administered in a saline solution at a rate of 0.035 mL min^−1^ through the jugular vein. The depth of anesthesia was controlled by testing the lack of response to stimulation of posterior limbs and by visual observation of a stable and regular breathing. After a 45‐min period of equilibration, three blood samples of 0.4 mL each were taken from the carotid artery at the midtime of three simultaneous 30 min urine collection. Volume of blood samples and fluid losses during surgery were replaced by corresponding amounts of normal saline solution. Inulin and paraaminohippurate were determined in plasma and urine samples as described below. Filtered fraction (FF) was calculated as the ratio GFR/RPF.

### Analytical determinations

#### Analysis of urine and plasma samples

Diuresis, urinary sodium, and plasma electrolyte concentration were determined by gravimetry and flame photometry, respectively, whereas inulin and paraaminohippurate were determined in plasma and urine samples by conventional methods (Smith et al. 1945; Young and Raisz 1952). Urinary sodium excretion was calculated as U_Na+_V.

DA was extracted from urine samples using alumina, separated by reverse‐phase high‐pressure liquid chromatography using a 4.6 × 150 mm, 5 *μ*m C18 column (Agilent Life Sciences and Chemical Analysis, Santa Clara, CA) and quantified amperometrically by a triple‐electrode system (ESA, Bedford, MA) (Eisenhofer et al. 1986).

Blood samples were also taken to determine plasma aldosterone levels by competitive radioimmunoassay using a commercial kit provided by Immunotech, Prague, Czech Republic.

#### Nicotinamide adenine dinucleotide phosphate diaphorase (NADPH‐d) reaction and NOS I immunohistochemistry

Animals were deeply anesthetized as described above and transcardially perfused with normal saline solution followed by fixative solution (4% paraformaldehyde in 0.1mol/L phosphate buffer, pH 7.4). Kidneys were removed and immersed in fixative solution at 4°C for 2 h postfixation. Transversal 40 *μ*m thick sections were cut with a Vibratome^®^ Series 1000 device.

NADPH‐d activity was determined according to a modified assay from Hope et al. (1991). Free‐floating 40 *μ*m thick kidney sections were incubated in 0.1 mol/L phosphate buffer (PB), pH 7.4/1% Triton X‐100/0.02% nitro blue tetrazolium/0.1% *β*‐NADPH at 37°C for 1 h. In order to avoid differences in NADPH‐d reactivity due to the staining procedure, sections from control and treated animals were incubated simultaneously. Then, sections were washed in PB, dehydrated, quickly cleared by xylene and coverslipped.

For immunohistochemical assay endogenous peroxidase activity was blocked with 1% hydrogen peroxide in PB for 30 min. Then, free‐floating sections were incubated with blocking solution containing 10% normal goat serum in phosphate buffered saline (PBS), pH 7.4, for 1 h. NOS I immunoreactivity was detected using a polyclonal rabbit antibody (dilution 1:3000) produced at the Cajal Institute (Rodrigo et al. 2001). The antibody was incubated overnight at 4°C and the specificity was corroborated in negative control sections. Immunoreactivity was visualized with a biotinylated goat antirabbit IgG (1:100; Sigma‐Aldrich), developed with ABC kit (Vector Laboratories, Burlingame, CA) and 0.03% 3,3′‐diaminobenzidine (Sigma‐Aldrich), 3% nickel ammonium sulfate (Merck, Darmstadt, Germany), and 0.01% hydrogen peroxide diluted in 0.1 mol/L buffer acetate, yielding a black product. Sections were dehydrated, cleared, and mounted.

Photographs of NADPH‐d reaction and NOS I immunoreactivity were taken with a Zeiss Axiophot microscope by an independent observer. To measure macula densa staining with NADPH‐d, five rats per group were used and 20 macula densa were analysed from each rat. Staining was measured in a 256 grey scale with background subtraction using the NIH Image software.

#### Tissue processing for Western blot analysis

Animals were deeply anesthetized as described above. Immediately after the animals were euthanized their kidneys were isolated. The left kidney was used to measure NO metabolites and the right kidney was used for western blots. The renal cortex was dissected and homogenized at 3000 rpm in an appropriate buffer (in mmol/l: 250 sucrose, 1 EDTA, 0.1 PMSF and 10 Tris‐ClH)**,** pH 7.6. Large tissue debris and nuclear fragments were removed by a low‐speed spin (1000 g, 10 min, 4°C). Protein concentration was measured using BCA^™^ Protein Assay Kit (Pierce, Rockford, IL). Absorbance for protein concentration measurement was read using an RT‐2100C microplate reader (Rayto, China) at 560 nm.

#### Western blots of NOS I and NOS III

Immunoblotting analysis was used to identify NOS I and NOS III. Aliquots of samples containing 50 *μ*g of proteins were loaded on 7.5% SDS polyacrylamide gels and then blotted on to PVDF membranes (GE Healthcare, Amersham Hybond‐P) at 60 V for 3 h. The membranes were washed in TBST buffer (50 mmol/l Tris‐ based saline, pH 7.4, containing 0.1% Tween 20) and blocked with 7,5% skimmed milk in TBST for 1 h. Blots were incubated overnight at 4°C with the NOS I antibody (polyclonal rabbit anti‐rat; diluted 1:500; Santa Cruz Biotechnology, Dallas, Texas), which recognized a 155‐kDa band, or with the NOS III antibody (polyclonal rabbit anti‐rat; diluted 1:250; BD Transduction Laboratories, San Jose, California) which recognized a 140‐kDa band. Beta‐tubulin was used as loading control (rabbit anti‐rat beta‐tubulin, 50‐kDa band; Abcam Inc., Cambridge, MA). The membranes were then incubated with a donkey anti‐rabbit IgG horseradish peroxidase‐conjugated secondary antibody (1:3000) (Abcam Inc., Cambridge, MA). Blots were visualized using a quimioluminiscent substrate Bio‐Lumina (from Kalium Technologies, BA, Argentina).

The relative protein levels were determined by analyzing the bands with Gel Pro Analyzer 3.1 for Windows and relative protein expression was calculated as the ratio NOS I (or NOS III)/*β*‐tubulin.

#### Preparation of tissue samples for determination of nitrates and nitrites

The renal cortex was dissected, homogenized in PBS pH 7.4 prepared with deionized water and centrifuged at 10,000 g for 20 min. The supernatant was ultracentrifuged at 100,000 g for 30 min. Then the new supernatant was filtered using a 10 kDa cut off filter (Amicon, EMD Millipore Corporation, Billerica, MA). The filters were rinsed with deionized water prior to ultrafiltration. A volume of 40 *μ*L of the filtrate was used to determine nitrates and nitrites concentration.

#### Nitrates and nitrites determination

The concentration of NO metabolites (nitrites and nitrates; NOx) in the ultrafiltrates of cortical tissue samples was determined according to the procedure described by Verdon et al. (1995).

#### Statistical analysis of data

Results are expressed as the mean ± SEM. Two‐way ANOVA was used to analyze the data, where one factor was the treatment (control or SCH 23390) and the other the diet (normal salt or high salt). The main effect of each factor was tested as well as the interaction within both factors. Bonferroni′s post hoc test was used for multiple comparisons when interaction was statistically significant, in which case the main effect of each factor was not informed (as each factor is influenced by the other) and simple main effects were informed separately. The analysis was performed using Graph Pad Prism version 5.0 for Windows, Graph Pad Software (San Diego, CA). The null hypothesis was rejected when *P* < 0.05.

## Results

### Renal function

Table 1 shows the results of renal function in rats under different sodium intake and treatment at the end of the study (day 5th). Water intake was increased in HS groups compared to NS groups (*P* < 0.001). SCH 23390 treatment showed no effect on drinking rate. Rats on a high salt intake (HS) showed a significantly higher diuresis than NS group (*P* < 0.001). Sodium excretion was also greater in HS than in NS (*P* < 0.001). Treatment with the specific D_1_‐like receptor antagonist SCH 23390 significantly reduced diuresis and natriuresis in the HS+SCH group when compared to HS. SCH 23390 did not alter volume or sodium excretion in the NS+SCH group compared to NS group. Plasma electrolytes remained stable in all groups and were not altered by SCH 23390 treatment (Table 1).

**Table 1:** Renal parameters in rats under normal or high salt intake with or without D_1_ receptor blockade

Renal parameters	NS	HS	NS + SCH	HS + SCH
Water intake (mL day^−1^)	15.59 ± 0.85	26.54 ± 2.18^a^	13.5 ± 0.85	25.67 ± 1.43^a^
Plasma Na+ (mmol L^−1^)	139.5 ± 1.5	142.33 ± 1.86	140 ± 1.8	141 ± 2.1
Plasma K+ (mmol L^−1^)	3.15 ± 0.05	3.7 ± 0.36	3.25 ± 0.07	3.5 ± 0.45
Diuresis, (mL day^−1^ 100 g bwt^−1^)	3.55 ± 0.19	11.67 ± 1.54^a^	3.13 ± 0.11	3.97 ± 0.49^d^
Urinary Na^+^ excretion, (mmol day^−1^ 100 g bwt^−1^)	0.44 ± 0.09	3.95 ± 0.24^a^	0.39 ± 0.06	1.32 ± 0.20^b^^,^ d
Urinary Dopamine, (ng day^−1^ 100 g bwt^−1^)	679 ± 24	1504 ± 94^a^	670 ± 34	1562 ± 89^c^

Results are expressed as the mean ± SEM (*n *=* *7). Two‐way ANOVA showed a statistically significant interaction (*P* < 0.001) between the effects of HS and SCH treatment on diuresis and urinary Na^+^ excretion. High salt intake had a significant overall effect on urinary dopamine (*P* < 0.001). NS, normal salt intake; HS, high salt intake; SCH: D1‐like receptor antagonist SCH 23390. Bonferroni post‐test as follows:

a *P* < 0.001 versus NS.

b *P* < 0.01 versus NS+SCH.

c *P* < 0.001 versus NS+SCH.

d *P* < 0.001 versus HS.

**Figure 1:**
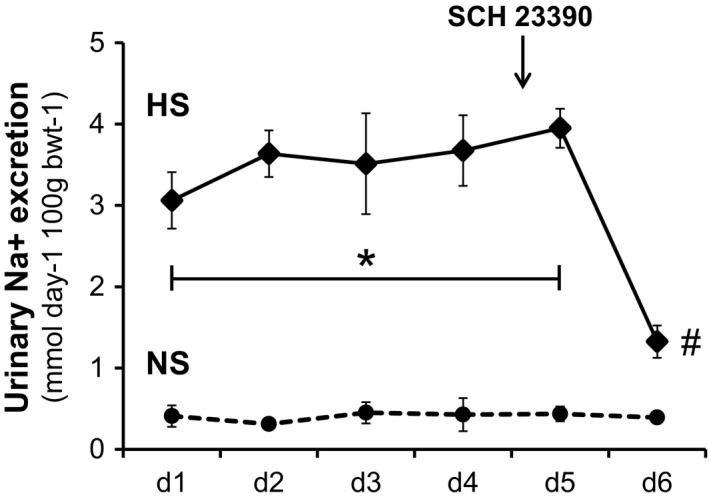
The different dynamic profile of urinary sodium excretion (mmol/day/100 g bwt) between normal salt (NS, dashed line) and high salt (HS, continuous line) groups before and after treatment with the D_1_‐like receptor antagonist SCH 23390. Horizontal axis shows days of urine collection as d1 to d6. Results are expressed as the mean ± SEM,* n *=* *7 rats per group. ANOVA for repeated measures. Symbols denote *P < 0.001 HS versus NS and #*P* < 0.001 HS post SCH23,390 versus HS.

The different dynamic profile of sodium excretion between NS and HS groups before and after treatment with SCH 23390 is shown in Figure 1. As expected, daily urinary sodium excretion was significantly higher –as much as six times– in HS than in NS group during the 5 days of the experiment (*P* < 0.001). On the other hand, the effect of SCH 23390 administration was quite different in either group. While SCH 23390 had no effect on urinary sodium excretion in the NS group, it sharply decreased urinary sodium excretion at 60% in the HS group (*P* < 0.001 HS+SCH vs. HS).

Urinary Dopamine (U_DA_V) markedly increased with high sodium intake (Table 1, *P* < 0.001, HS vs. NS rats). SCH 23390 treatment did not modify U_DA_V neither in the NS nor in the HS rats. Plasma aldosterone levels were significantly reduced by high salt intake from 284 ± 24 in NS group to 133 ± 15 pg ml^−1^ in HS rats, *P* < 0.05. The increase in U_DA_V and the decrease in plasma aldosterone show the effectiveness of neurohumoral response to HS intake compared to NS intake.

### Renal perfusion

**Figure 2:**
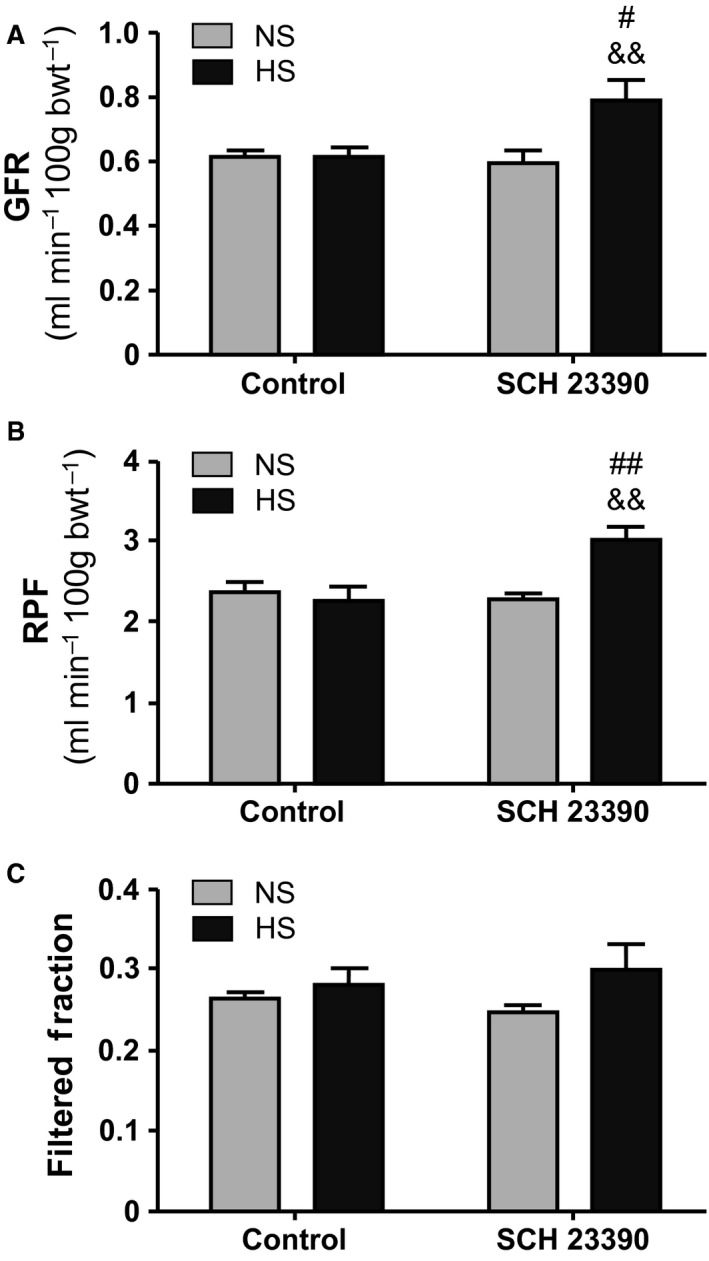
Renal hemodynamic parameters. NS, normal salt intake and HS, high salt intake. SCH 23390, D_1_‐like receptor antagonist. Results are expressed as mean ± SEM (*n *=* *7). (A) GFR, Glomerular Filtration Rate (mL/min/100 g bwt). Two way ANOVA showed a statistically significant interaction (*P* < 0.05) between the effects of HS and SCH 23390 treatment. # *P* < 0.05 versus NS+SCH; && *P* < 0.01 versus HS. (B) RPF, Renal Plasma Flow (ml/min/100 g bwt). Two way ANOVA showed a statistically significant interaction (*P* < 0.01) between the effects of HS intake and SCH 23390 treatment. ## *P* < 0.01 versus NS+SCH; && *P* < 0.01 versus HS. (C) Filtered Fraction (GFR/RPF). Two way ANOVA did not show a statistically significant interaction nor differences between the effects of HS and SCH 23390 treatment.

RPF, GFR, and FF are shown in Figure 2. NS and HS groups had similar values of RPF, GFR, and FF. Treatment with the specific D_1_‐like receptor antagonist SCH 23390 caused a significant change in renal perfusion in the HS+SCH group. Both RPF and GFR significantly increased (*P* < 0.01 vs. their respective HS groups, Fig. 2A and B) while filtered fraction remained unchanged (Fig 2C). SCH 23390 treatment did not affect renal hemodynamic parameters in NS+SCH rats compared with NS rats.

Systolic blood pressure was (mmHg) 110 ± 2 in NS, 105 ± 4 in HS and 109 ± 3 in NS+SCH groups and significantly increased to 117 ± 3 in the HS+SCH group (*P* < 0.05 vs. HS group). However, the increment in blood pressure in the HS+SCH group is within the autoregulatory range of renal perfusion.

### Histochemistry and immunohistochemistry

**Figure 3:**
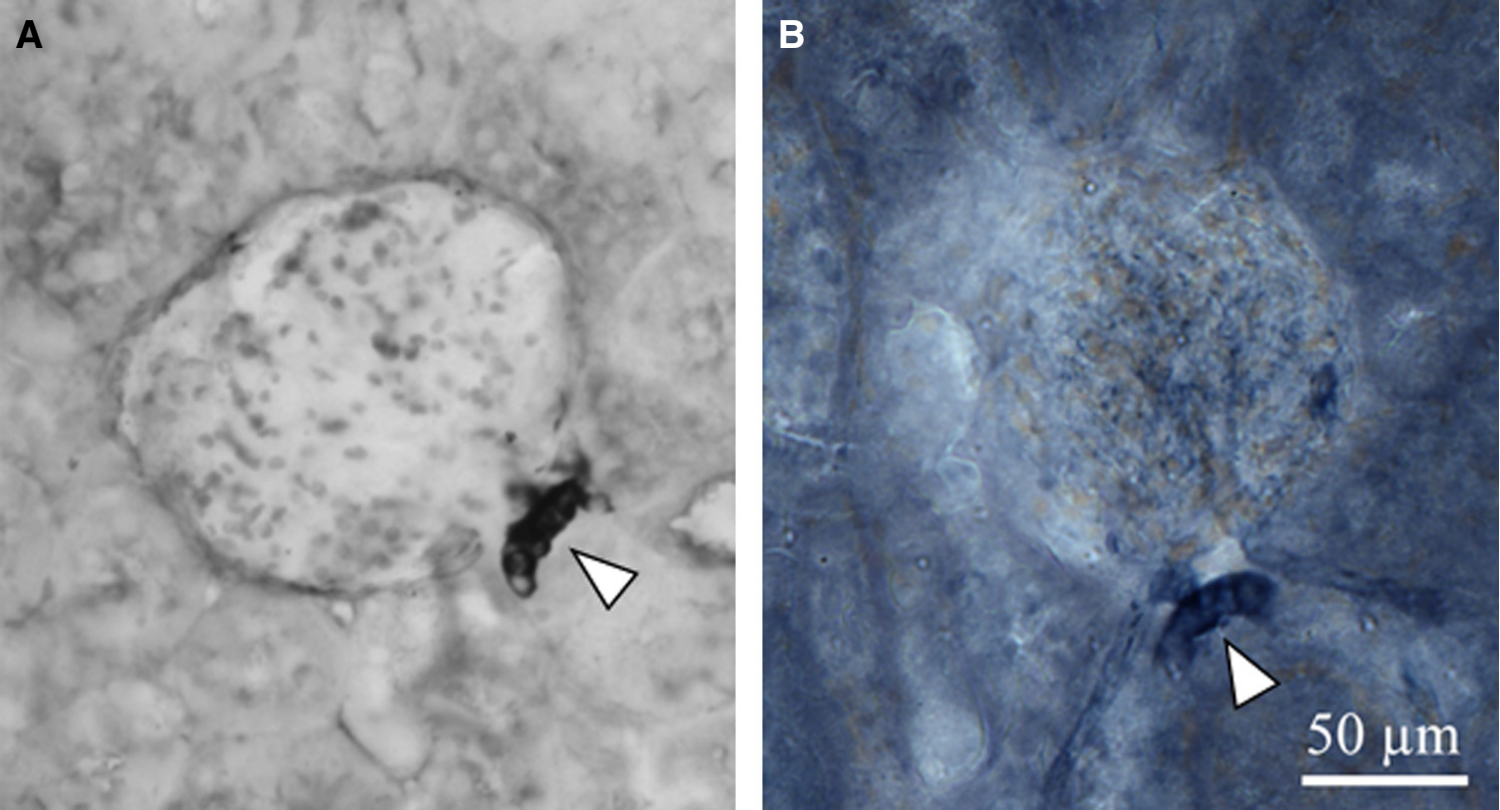
Representative microphotographs of (A) NOS I immunoreactivity (IHC, immunohistochemistry) and (B) NADPH‐d reaction. Arrowheads show the macula densa staining.

**Figure 4:**
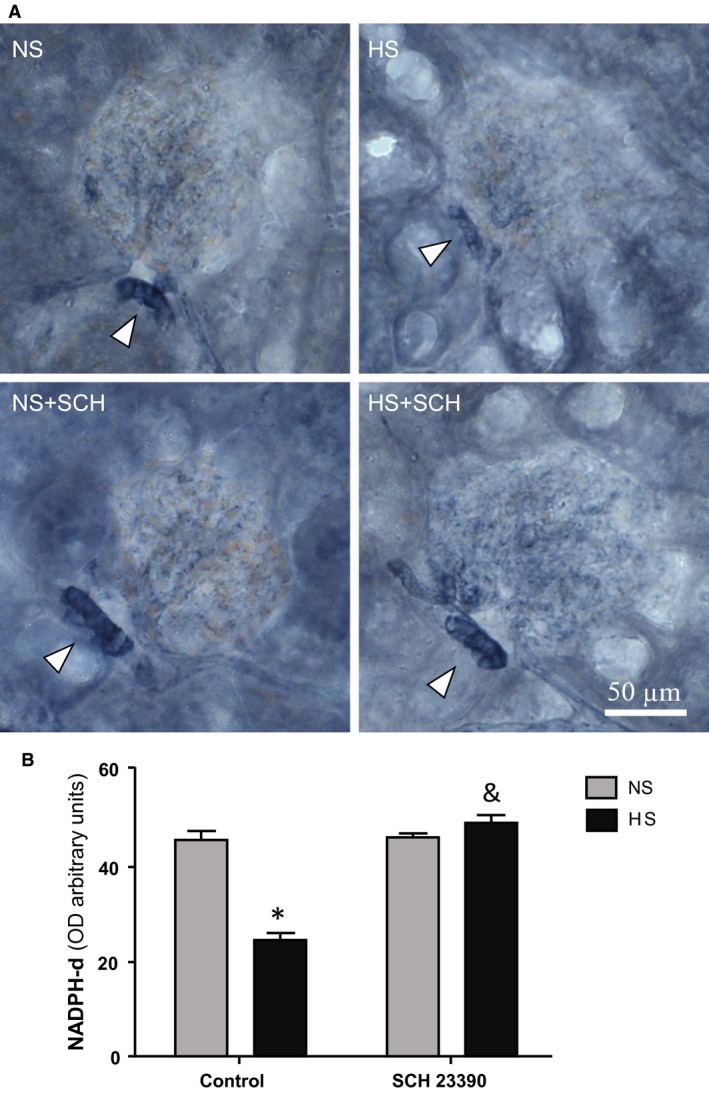
NADPH diaphorase (NADPH‐d) staining in renal cortical tissue. (A) Representative images of NADPH‐d staining. NS: control rats receiving a normal salt diet that were not treated with D_1_‐like receptor antagonist SCH 23390; HS: control rats receiving a high salt diet that were not treated with D_1_‐like receptor antagonist SCH 23390; NS+SCH: rats receiving a normal salt diet and treated with D_1_‐like receptor antagonist SCH 23390; HS+SCH: rats receiving a high salt diet and treated with D_1_‐like receptor antagonist SCH 23390. Arrowheads point to macula densa staining. (B) NADPH‐d optical density analysis. Two way ANOVA showed a statistically significant interaction (*P* < 0.001) between the effects of high salt intake and SCH 23,390 treatment. * *P* < 0.001 versus NS; & *P* < 0.001 versus HS. Results are expressed as mean ± SEM; (*n *=* *5).

As observed in Figure 3, both the antibody against NOS I (A) and NADPH‐d reaction (B) show MD cells stained at the same region confronting the vascular pole of the respective glomerulus but no other cortical structures (Fig. 3, arrows). These results in renal cortex are in line with those reported by other authors (Mundel et al. 1992; Tojo et al. 1994; Ichii et al. 2008). We then analyzed whether NADPH‐d activity was sensitive to variations in sodium intake and to D_1_‐like receptor blockade. Figure 4 shows that NADPH‐d activity in NS group was intense and neat (Fig. 4A: NS, arrow) in density units 46.10 ± 1.80 (Fig. 4B, bottom bars). Rats on HS intake, instead, had a significantly weaker NADPH‐d signal than NS group (Fig. 4A: HS, arrows) 24.76 ± 4.64 as recorded by optical density (*P* < 0.001 vs. NS, Fig. 4B, bottom bars). When DA effect was interrupted by the D_1_‐like receptor antagonist, NADPH‐d activity in MD cells from HS group regained intensity similar to NS group (Fig. 4A: HS+SCH, arrow). Then, NADPH‐d optical density in HS+SCH rats increased to 49.27 ± 1.92 (*P* < 0.001 vs. HS, Fig. 4B, bottom bars). NADPH‐d activity in MD cells of NS rats was not modified by SCH 23390 (Fig. 4A: NS+SCH, arrow, optical density 46.42 ± 2.35, Fig. 4B, bottom bars).

### NOS I and NOS III expression by Western blot

**Figure 5:**
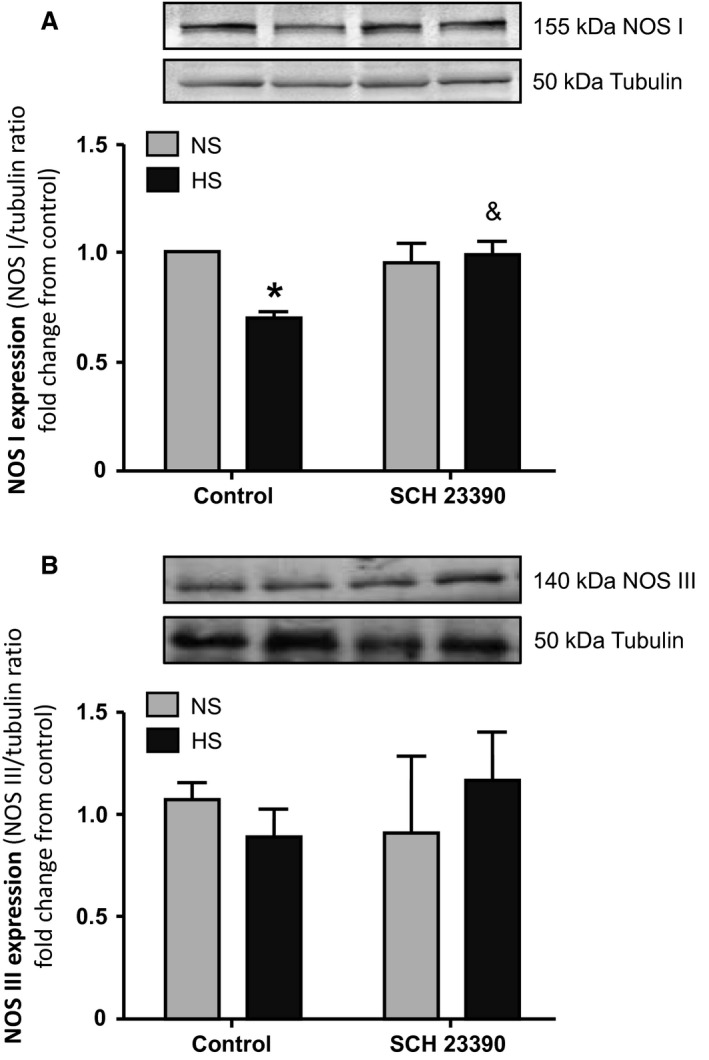
(A) NOS I expression and (B) NOS III expression in homogenates of renal cortex. NS: normal sodium intake; HS: high sodium intake; control: rats not treated with the D_1_‐like receptor antagonist SCH 23390; SCH 23390: rats treated with the D_1_‐like receptor antagonist. (A) Top panel: representative western blot of NOS I (155 kDa band) and tubulin (50 kDa band) in renal cortex. Bottom bars: NOS I expression indicated as NOS I/tubulin ratio fold change from NS control rats. (B) Top panel: representative western blot of NOS III (140 kDa band) and tubulin (50 kDa band) in renal cortex. Bottom bars: NOS III expression indicated as NOS III/tubulin ratio fold change from NS control rats. (A) Two‐way ANOVA showed a statistically significant interaction (*P* < 0.05) between the effects of high salt intake and SCH 23390 treatment on NOS I expression. * *P* < 0.01 control HS rats versus control NS rats; & *P* < 0.05 HS+SCH versus HS. Results are expressed as mean ± SEM (*n *=* *5). (B) Two‐way ANOVA did not show statistical differences in NOS III expression. Results are expressed as mean ± SEM (*n *=* *5).

Figure 5 shows WB experiments of NOS I expression (A) and NOS III expression (B) in rats on NS and HS intake whether or not treated with SCH 23390. As it can be seen, in control rats HS intake induced a significant reduction in NOS I expression when compared with NS rats (*P* < 0.05). When the D_1_‐like receptor was blocked by SCH 23390, results of NOS I expression were different depending on the rats′ sodium intake. In HS group, treatment with SCH 23390 restored NOS I expression to similar levels as those observed in NS rats. However, in NS rats, NOS I expression did not show variations upon SCH 23390 treatment. On the contrary NOS III expression (Fig. 5B) was not modified by sodium intake or SCH 23390 treatment.

### NOx determination in cortical homogenates

**Figure 6:**
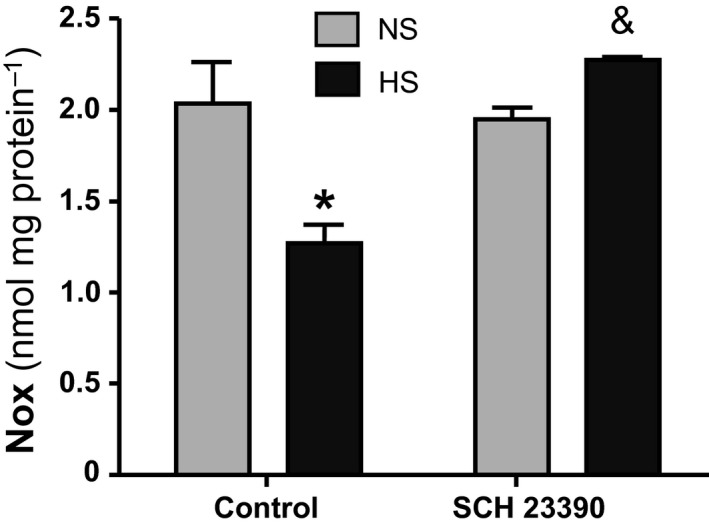
NO metabolites (Nitrates and Nitrites, NOx) production in renal cortex. NS: normal sodium intake; HS: high sodium intake; control: rats not treated with the D_1_‐like receptor antagonist SCH 23390; SCH 23390: rats treated with the D_1_‐like receptor antagonist. Two way ANOVA showed a statistically significant interaction (*P* < 0.01) between the effects of high salt intake and SCH 23390 treatment on NOx production in renal cortex. *P < 0.01 HS versus NS; & *P* < 0.001 HS + SCH 23390 versus HS. Results are expressed as mean ± SEM (*n *=* *5).

To analyze whether variations in NADPH‐d activity and NOS I expression were paralleled by changes in NO synthesis, the concentration of NOx was determined in renal cortical homogenates. Basal NOx production in control NS group was 2.04 ± 0.22 nmol mg protein^−1^. When rats had a HS intake, NOx significantly decreased to 1.28 ± 0.10 nmol mg protein^−1^ (*P* < 0.01 vs. NS). The decrease in NOx production was reverted in HS group by D_1_‐like receptor blockade to 2.27 ± 0.02 nmol mg protein^−1^ (*P* < 0.01 HS+SCH vs. HS rats). No effect of SCH 23390 on NOx levels was observed in the NS+SCH group compared to control NS group. These results can be observed in Figure 6.

## Discussion

In this study rats under high sodium intake showed the expected changes in several variables of renal function and hormonal profile when compared with rats on a normal sodium intake. High sodium intake is accompanied by an increase of renal dopamine synthesis and release in epithelial cells of the proximal tubule and an increase in urinary dopamine which results in decreased sodium reabsorption recorded as an increment in diuresis and natriuresis. On the other hand, in MD cells, a more distal structure of the nephron, HS is associated with a significant decrease in NOS I expression, a lower NADPH‐d signal, together with a reduction in cortical NOx production. There are some controversies between cortical NOS I expression and NO production during HS intake. In contrast, some authors have found that NO production is increased during HS intake (Lu et al. 2010). These discrepancies may be explained by the amount of NaCl intake. Lu et al. used a high‐salt diet (8% NaCl) with 0.45% NaCl in the drinking water while we used 1% NaCl in drinking water and standard diet. Another difference is the rat strain. They used Sprague‐Dawley while we used Wistar rats. Although we did not find studies comparing Sprague‐Dawley and Wistar rats concerning NO production and different amounts of sodium loading, various strain differences have been reported in other fields like renal failure (Saracyn et al. 2012), insulin sensitivity during pregnancy (Cacho et al. 2008) and global cerebral ischemia (Fuzik et al. 2013). Therefore we cannot discard strain differences. Besides, the discrepancies may be related, at least in part, to the use of NOS I antibody that recognizes different NOS I isoforms. NADPH‐d staining is widely employed to detect NOS‐containing cells in neural and non‐neural tissues. With the appropriate fixation procedure, this method can detect cells containing any of the NOS isoforms (Gonzalez‐Zulueta et al. 2001). The NADPH‐d reaction can be used to monitor NOS activity at a cellular level of resolution (Morris et al. 1997). Bearing in mind that MD cells express the NOS I isoform, our present findings led us to suggest that NADPH‐d signal intensity parallels changes in NOS I expression and activity, such as NOx production. This finding is further supported by the fact that NOS III expression remained unaltered by the increase in sodium intake. Such a changing scenario upon HS intake has also prompted us to speculate whether the effects related to high DA coming from PT cells and those in MD related to a decrease in NO production are independent mechanisms of an adaptive response to HS intake or whether they may have an interrelationship to regulate renal plasma flow and glomerular filtration rate. To test this hypothesis, both NS and HS rats were treated with the specific D_1_‐ like receptor antagonist SCH 23390 in order to block renal dopamine effects. It is accepted that renal tissues express all different types of dopamine receptors and MD cells express D_1_R and D_3_R (Zeng et al. 2004).

In our study, SCH 23390 significantly reduced diuresis and natriuresis in HS rats. There was an almost 60% decrease in hydroelectrolyte excretion after D_1_‐like receptor blockade (Table 1 and Fig. 1). Water intake was not modified by SCH 23390 treatment. Together with the decrease in hydroelectrolyte excretion in HS rats, treatment with the D_1_‐like receptor antagonist was accompanied by significant changes in the expression of NOS I, NADPH‐d, and NOx production increasing all back to control levels (Figs. 3 to 6). RPF and GFR were also significantly increased by SCH 23390 (Fig. 2). Thus, after D_1_‐like receptor blockade all changes observed under HS intake were reversed and this effect resulted in an increment of renal perfusion above the levels observed in NS and HS groups. SCH 23390 had no effect on the same variables in the NS group. NOS III expression did not change upon SCH 23390 treatment regardless of sodium intake.

Concerning the explanation about the results described here, MD cells express the Na/K/2Cl cotransporter (NKCC2), K^+^ channel (ROMK) and the Na/H exchanger at the apical membrane (Bell and Lapointe 1997; Edwards et al. 2014). NKCC2 in MD cells is regarded as a sensor for changes in Na^+^ and Cl^−^ concentrations, especially chloride, in the tubule lumen. NKCC2 modifies the amount of Cl^−^ that enters MD cells depending upon how much NaCl reaches the distal portions of the nephron in order to signal for the necessity to activate TGF. When the concentration of NaCl increases TGF is activated, the AA is constricted (Schnermann 2003) and NO production is reduced in MD cells. It has been described that under conditions of sodium load TGF is attenuated and that DA could play a role in TGF inhibition (Schnermann et al. 1990; Häberle and Königbauer 1991) because DA has a vasodilator effect. The role of DA, however, may be more complex than expected as a vasodilator. Dopamine inhibits NaCl reabsorption in the thick ascending limb of Henle′s loop by decreasing NKCC2 activity (Grider et al. 2003; Wang et al. 2010). Thus, the amount of NaCl that reaches MD cells increases and this increment should activate TGF which is quite the opposite as stated above. On the other hand, inhibition of the NKCC2 cotransporter is part of the DA natriuretic effect. In MD cells, particularly, inhibition of NKCC2 transport is sensed as a decrease in sodium intake and this effect could contribute to TGF attenuation. Besides, the vasodilator effect of DA on renal vessels including glomeruli is not observed with renal dopamine (Siragy et al. 1989; Wang et al. 1997) which is the dopamine described in this work, but instead with neural and injected dopamine (Baines and Drangova 1986; Schnermann et al. 1990). Increase in diuresis and natriuresis are the well‐known effects of renal dopamine (Wang et al. 1997).

In line with our results, studies performed in central nervous system in models of Parkinson′s disease (Yuste et al. 2012) or behavior (Tanda et al. 2009) seem to show a negative reciprocity between DA and NO. When NO decreases, DA activity is increased and vice versa (Sammut et al. 2007). Present results do not allow to speculate on a direct regulation of DA on NOS I activity, or to rule out other intermediary phosphoproteins, like DARPP 32 in Henle′s loop which can intermediate between DA and NOS (Yuste et al. 2012) but rather to consider that changes in NOS I, NADPH‐d, NOx production and renal hemodynamic are the consequence of a sharp decrease in NaCl reaching MD cells after D_1_‐like receptor blockade. Nephron segments upstream MD cells reabsorb almost 90% of filtered NaCl and all the sodium transporters located in the apical or basolateral membranes of those segments are inhibited by DA (Aperia 2000; Harris and Zhang 2012). In Figure 1 it can be seen that sodium excretion under HS intake was several times higher than in NS rats during all the days that the HS rats received 1% NaCl. But when SCH 23390 treatment was started, sodium and volume excretion sharply decreased in HS rats. A similar consideration regarding the relationship between renal dopamine and COX2 expression in MD cells has been reported by Zhang et al. (2005). It is therefore more likely that MD cells, sensing an abrupt decrease in luminal NaCl concentration with SCH 23390 treatment, responded as if the condition had changed from a HS intake to a much lower salt intake and, then, levels of NOS I and NOx were restored to blunt the relative AA constriction. According to other reports, it is also probable that NKCC2 expression shifted from the A isoform to the B isoform after SCH 23390 treatment. NKCC2 B has a higher affinity for Cl^−^ than NKCC2 A isoform and is modulated by low salt intake (Schiessl et al. 2013).

The action on renal function described here seems to be specific to D_1_‐like receptor. Blockade of D_2_ receptor on RPF has been described in humans by MacDonald (1991) and Bughi et al.(1994). In these studies researchers reported that RPF remained unaltered in individuals under high salt intake treated with the specific D_2_ receptor blocker domperidone.

The mild increase in SBP observed in HS+SCH rats is probably due to retained fluids since rats’ diuresis and natriuresis fell markedly while water intake did not change. Fluid retention expands extracellular fluid volume and, as a consequence, blood pressure increases. Models of salt‐sensitive hypertension display a similar pattern as observed in Bradykinin‐B_2_ receptor KO mice (Alfie et al. 1997) and in rats or mice with disruption or alteration in the expression or activity of dopamine D_1_R (Harris and Zhang 2012; Di Ciano et al. 2015).

Since the increase in SBP was mild it should be expected to be within the autoregulatory range of RPF. In line with this, mice lacking Bradykinin‐B_2_ receptors become hypertensive upon a high sodium intake. Despite that the increase in blood pressure is higher than in the present study, RPF and GFR remain stable compared to animals on a NS intake (Alfie et al. 1997). However, we cannot rule out that the combination of HS intake, which induces several hormonal changes, plus the blockade of D_1_R receptor may shift autoregulation of RPF.

The effect of D_1_‐like receptor blockade by SCH 23390 can be seen as paradoxical or unexpected. However, other reports come in support of present findings: renal sites of SCH 23390 binding are predominantly tubular epithelial cells, including MD cells and not vascular structures (Ricci et al. 1993), and it has also been shown that SCH 23390 administered in the renal artery in dogs with a moderate sodium repletion decreases hydroelectrolyte excretion by 50% but does not affect renal perfusion (Siragy et al. 1989).

In summary, renal dopamine synthesized in proximal tubules and NO released by the activity of NOS I in MD cells coordinately regulate RPF and GFR under HS intake. Since the increase in renal DA production is secondary to a HS intake, findings give an insight into a mechanism which links a systemic effect to a local regulatory mechanism such as NO release from MD cells and its effect on AA diameter. Ultimately, the negative relationship between DA and NOS I regulates the NaCl load on thick ascending limb cells transport capacity and stabilizes the delivery of NaCl to the distal nephron.

## Conflict of Interest

The authors declare that they have no conflict of interest.

## Compliance with Ethical Standards

All procedures performed in studies involving animals were in accordance with the ethical standards of European Convention for the Protection of Vertebrate Animals used for Experimental and other Scientific Purposes and the institution or practice at which the studies were conducted.

## References

[phy213202-bib-0001] Alfie, M. E. , D. H. Sigmon , S. I. Pomposiello , and O. A. Carretero . 1997 Effect of high salt intake in mutant mice lacking bradykinin‐B2 receptors. Hypertension 29:483–487. 9039146 10.1161/01.hyp.29.1.483

[phy213202-bib-0002] Aperia, A. C. 2000 Intrarenal dopamine: a key signal in the interactive regulation of sodium metabolism. Annu. Rev. Physiol. 62:621–647. 10845105 10.1146/annurev.physiol.62.1.621

[phy213202-bib-0003] Arima, S. , and S. Ito . 2000 Isolated juxtaglomerular apparatus as a tool for exploring glomerular hemodynamics: application of microperfusion techniques. Exp. Nephrol. 8:304–311. 10940731 10.1159/000020683

[phy213202-bib-0004] Baines, A. D. , and R. Drangova . 1986 Neural not tubular dopamine increases glomerular filtration rate in perfused rat kidneys. Am. J. Physiol. 250:F674–F679. 2421588 10.1152/ajprenal.1986.250.4.F674

[phy213202-bib-0005] Bell, P. D. , and J. Y. Lapointe . 1997 Characteristics of membrane transport processes of macula densa cells. Clin. Exp. Pharmacol. Physiol. 24:541–547. 9248675 10.1111/j.1440-1681.1997.tb01243.x

[phy213202-bib-0006] Bughi, S. , E. Jost‐Vu , I. Antonipillai , J. Nadler , and R. Horton . 1994 Effect of dopamine2 blockade on renal function under varied sodium intake. J. Clin. Endocrinol. 78:1079–1084. 10.1210/jcem.78.5.8175964 8175964

[phy213202-bib-0007] Cacho, J. , J. Sevillano , J. de Castro , E. Herrera , and M. P. Ramos . 2008 Validation of simple indexes to assess insulin sensitivity during pregnancy in Wistar and Sprague‐Dawley rats. Am. J. Physiol. Endocrinol. Metab. 295:E1269–E1276. 18796548 10.1152/ajpendo.90207.2008

[phy213202-bib-0008] Carlström, M. , C. S. Wilcox , and W. J. Arendshorst . 2015 Renal autoregulation in health and disease. Physiol. Rev. 95:405–511. 25834230 10.1152/physrev.00042.2012 PMC4551215

[phy213202-bib-0009] Chen, C. J. , and M. F. Lokhandwala . 1992 An impairment of renal tubular DA‐1 receptor function as the causative factor for diminished natriuresis to volume expansion in spontaneously hypertensive rats. Clin. Exp. Hypertens. 14:615–628. 10.3109/10641969209036211 1352742

[phy213202-bib-0010] De Luca Sarobe, V. , S. Nowicki , A. Carranza , G. Levin , M. Barontini , E. Arrizurieta , et al. 2005 Low sodium intake induces an increase in renal monoamine oxidase activity in the rat. Involvement of an angiotensin II dependent mechanism. Acta Physiol. Scand. 185:161–167. 16168010 10.1111/j.1365-201X.2005.01473.x

[phy213202-bib-0011] De Luca Sarobe, V. , L. Di Ciano , A. M. Carranza , G. Levin , E. E. Arrizurieta , and F. R. Ibarra . 2010 Patterns of renal dopamine release to regulate diuresis and natriuresis during volume expansion. Role of renal monoamine‐oxidase. Medicina (B Aires) 70:60–64. 20228026

[phy213202-bib-0012] Di Ciano, L. A. , P. J. Azurmendi , C. Colombero , G. Levin , E. M. Oddo , E. E. Arrizurieta , et al. 2015 Defective renal dopamine function and sodium‐sensitive hypertension in adult ovariectomized Wistar rats: role of the cytochrome P‐450 pathway. Am. J. Physiol. Renal. Physiol. 308:F1358–F1368. 25925257 10.1152/ajprenal.00450.2014

[phy213202-bib-0013] Edwards, A. , H. Castrop , K. Laghmani , V. Vallon , and A. T. Layton . 2014 Effects of NKCC2 isoform regulation on NaCl transport in thick ascending limb and macula densa: a modeling study. Am. J. Physiol. Renal. Physiol. 307:F137–F146. 24848496 10.1152/ajprenal.00158.2014 PMC4101627

[phy213202-bib-0014] Eisenhofer, G. , D. S. Goldstein , R. Stull , H. R. Keiser , T. Sunderland , D. L. Murphy , et al. 1986 Simultaneous liquid‐chromatographic determination of 3,4‐dyhydroxyphenylglyicol, catecholamines and dyhydroxyphenylalanine in plasma and their responses to inhibition of monoamineoxidase. Clin. Chem. 32:2030–2032. 3096593

[phy213202-bib-0015] Fuzik, J. , L. Gellért , G. Oláh , J. Herédi , K. Kocsis , L. Knapp , et al. 2013 Fundamental interstrain differences in cortical activity between Wistar and Sprague‐Dawley rats during global ischemia. Neuroscience 228:371–381. 23103797 10.1016/j.neuroscience.2012.10.042

[phy213202-bib-0016] Gao, X. , A. Patzak , M. Sendeski , P. G. Scheffer , T. Teerlink , J. Sällström , et al. 2011 Adenosine A₁‐receptor deficiency diminishes afferent arteriolar and blood pressure responses during nitric oxide inhibition and angiotensin II treatment. Am. J. Physiol. Regul. Integr. Comp. Physiol. 301:R1669–R1681. 21975649 10.1152/ajpregu.00268.2011

[phy213202-bib-0017] Gonzalez‐Zulueta, M. , V. L. Dawson , and T. M. Dawson . 2001 Histochemical analysis of nitric oxide synthase by NADPH diaphorase staining. Curr. Protoc. Toxicol. Chapter 10: Unit 10.6. 10.1002/0471140856.tx1006s01 23045028

[phy213202-bib-0018] Grider, J. S. , C. E. Ott , and B. A. Jackson . 2003 Dopamine D1 receptor‐dependent inhibition of NaCl transport in the rat thick ascending limb: mechanism of action. Eur. J. Pharmacol. 473:185–190. 12892837 10.1016/s0014-2999(03)01965-4

[phy213202-bib-0019] Häberle, D. A. , and B. Königbauer . 1991 Inhibition of tubuloglomerular feedback by the D1 agonist fenoldopam in chronically salt‐loaded rats. J. Physiol. 441:23–34. 1687747 10.1113/jphysiol.1991.sp018736 PMC1180183

[phy213202-bib-0020] Hansell, P. , and A. Fasching . 1991 The effect of dopamine receptor blockade on natriuresis is dependent on the degree of hypervolemia. Kidney Int. 39:253–258. 2002639 10.1038/ki.1991.30

[phy213202-bib-0021] Harris, R. C. , and M. Z. Zhang . 2012 Dopamine, the kidney, and hypertension. Curr. Hypertens. Rep. 14:138–143. 22407378 10.1007/s11906-012-0253-z PMC3742329

[phy213202-bib-0022] Hope, B. T. , G. J. Michael , K. M. Knigge , and S. R. Vincent . 1991 Neuronal NADPH diaphorase is a nitric oxide synthase. Proc. Natl Acad. Sci. 88:2811–2814. 1707173 10.1073/pnas.88.7.2811 PMC51329

[phy213202-bib-0023] Ibarra, F. R. , T. Galcerán , E. Oddo , and E. Arrizurieta . 1998 Changes in glomerular filtration rate and renal plasma flow in cirrhotic rats during converting enzyme inhibition. Ren. Fail. 20:65–74. 9509561 10.3109/08860229809045090

[phy213202-bib-0024] Ibarra, F. R. , I. Armando , S. Nowicki , A. Carranza , V. De Luca Sarobe , E. E. Arrizurieta , et al. 2005 Dopamine is metabolised by different enzymes along the rat nephron. Pflugers Arch. 450:185–191. 15864503 10.1007/s00424-005-1386-6

[phy213202-bib-0025] Ichii, O. , A. Yabuki , T. Ojima , M. Matsumoto , K. Taniguchi , and S. Suzuki . 2008 Immunohistochemical localization of renin, NO synthase‐1, and cyclooxygenase‐2 in rodent kidney. Histol. Histopathol. 23:143–150. 17999370 10.14670/HH-23.143

[phy213202-bib-0026] Imig, J. D. , and R. J. Roman . 1992 Nitric oxide modulates vascular tone in preglomerular arterioles. Hypertension 19:770–774. 1592479 10.1161/01.hyp.19.6.770

[phy213202-bib-0027] Just, A. , and W. J. Arendshorst . 2005 Nitric oxide blunts myogenic autoregulation in rat renal but not skeletal muscle circulation via tubuloglomerular feedback. J. Physiol. 569:959–974. 16223765 10.1113/jphysiol.2005.094888 PMC1464274

[phy213202-bib-0028] Just, A. , and W. J. Arendshorst . 2007 A novel mechanism of renal blood flow autoregulation and the autoregulatory role of A1 adenosine receptors in mice. Am. J. Physiol. Renal. Physiol. 293:F1489–F1500. 17728380 10.1152/ajprenal.00256.2007

[phy213202-bib-0029] Lee, M. R. 1993 Dopamine and the kidney: ten years on. Clin. Sci. (Lond.) 84:357–375. 8482041 10.1042/cs0840357

[phy213202-bib-0030] Lu, D. , Y. Fu , A. Lopez‐Ruiz , R. Zhang , R. Juncos , H. Liu , et al. 2010 Salt‐sensitive splice variant of nNOS expressed in the macula densa cells. Am. J. Physiol. Renal. Physiol. 298:F1465–F1471. 20335319 10.1152/ajprenal.00650.2009 PMC2886819

[phy213202-bib-0031] MacDonald, T. M. 1991 Metoclopramide, domperidone and dopamine in man: actions and nteractions. Eur. J. Clin. Pharmacol. 40:225–230. 2060557 10.1007/BF00315200

[phy213202-bib-0032] Moore, L. C. , J. Schnermann , and S. Yarimizu . 1979 Feedback mediation of SNGFR autoregulation in hydropenic and DOCA‐ and salt‐loaded rats. Am. J. Physiol. 6:F63–F74. 10.1152/ajprenal.1979.237.1.F63 464061

[phy213202-bib-0033] Morris, B. J. , C. S. Simpson , S. Mundell , K. Maceachern , H. M. Johnston , and A. M. Nolan . 1997 Dynamic changes in NADPH‐diaphorase staining reflect activity of nitric oxide synthase: evidence for a dopaminergic regulation of striatal nitric oxide release. Neuropharmacology 36:1589–1599. 9517430 10.1016/s0028-3908(97)00159-7

[phy213202-bib-0034] Mundel, P. , S. Bachmann , M. Bader , A. Fischer , W. Kummer , B. Mayer , et al. 1992 Expression of nitric oxide synthase in kidney macula densa cells. *Kidney Int* 42, 1017‐9.Palmer, L.G. & Schnermann, J. 2015. Integrated Control of Na Transport along the Nephron. Clin. J. Am. Soc. Nephrol. 10:676–687. 10.1038/ki.1992.382 1280698

[phy213202-bib-0800] Palmer, L. G. , and J. Schnermann . 2015 Integrated control of Na transport along the nephron. Clin. J. Am. Soc. Nephrol. 10:676–687. 25098598 10.2215/CJN.12391213 PMC4386267

[phy213202-bib-0035] Persson, A. E. , and S. Bachmann . 2000 Constitutive nitric oxide synthesis in the kidney‐functions at the juxtaglomerular apparatus. Acta Physiol. Scand. 169:317–324. 10951123 10.1046/j.1365-201x.2000.00750.x

[phy213202-bib-0036] Reddy, S. , N. Salipan‐Moore , S. Mildenberger , N. Willis , and A. Z. Györy . 1998 Acute volume expansion and salt‐loading studies in rats. The role of atrial natriuretic peptide and catecholamines. Nephron 79:192–200. 9647500 10.1159/000045024

[phy213202-bib-0037] Ricci, A. , S. Escaf , J. A. Vega , and F. Amenta . 1993 Autoradiographic localization of dopamine D1 receptors in the human kidney. J. Pharmacol. Exp. Ther. 264:431–437. 8423542

[phy213202-bib-0038] Rodrigo, J. , D. Alonso , A. P. Fernández , J. Serrano , A. Richart , J. C. López , et al. 2001 Neuronal and inducible nitric oxide synthase expression and protein nitration in rat cerebellum after oxygen and glucose deprivation. Brain Res. 909:20–45. 11478918 10.1016/s0006-8993(01)02613-0

[phy213202-bib-0039] Sammut, S. , K. E. Bray , and A. R. West . 2007 Dopamine D2 receptor‐dependent modulation of striatal NO synthase activity. Psychopharmacology 191:793–803. 17206493 10.1007/s00213-006-0681-z

[phy213202-bib-0040] Saracyn, M. , J. Patera , J. Kocik , M. Brytan , R. Zdanowski , A. Lubas , et al. 2012 Strain of experimental animals and modulation of nitric oxide pathway: their influence on development of renal failure in an experimental model of hepatorenal syndrome. Arch. Med. Sci. 8:555–562. 22852015 10.5114/aoms.2012.29281 PMC3400905

[phy213202-bib-0041] Schiessl, I. M. , A. Rosenauer , V. Kattler , W. W. Minuth , M. Oppermann , and H. Castrop . 2013 Dietary salt intake modulates differential splicing of the Na‐K‐2Cl cotransporter NKCC2. Am. J. Physiol. Renal. Physiol. 305:F1139–F1148. 23946287 10.1152/ajprenal.00259.2013

[phy213202-bib-0042] Schnermann, J. 2003 Homer W. Smith Award lecture. The juxtaglomerular apparatus: from anatomical peculiarity to physiological relevance. J. Am. Soc. Nephrol. 14:1681–1694. 12761271 10.1097/01.asn.0000069221.69551.30

[phy213202-bib-0043] Schnermann, J. , K. M. Todd , and J. P. Briggs . 1990 Effect of dopamine on the tubuloglomerular feedback mechanism. Am. J. Physiol. 258:F790–F798. 2330978 10.1152/ajprenal.1990.258.4.F790

[phy213202-bib-0044] Selkurt, E. E. , P. W. Hall , and M. P. Spencer . 1949 Influence of graded arterial pressure decrement on renal clearance of creatinine, p‐aminohippurate and sodium. Am. J. Physiol. 159:369–378. 15393481 10.1152/ajplegacy.1949.159.2.369

[phy213202-bib-0045] Singh, I. , M. Grams , W. H. Wang , T. Yang , P. Killen , A. Smart , et al. 1996 Coordinate regulation of renal expression of nitric oxide synthase, renin, and angiotensinogen mRNA by dietary salt. Am. J. Physiol. 270:F1027–F1037. 8764322 10.1152/ajprenal.1996.270.6.F1027

[phy213202-bib-0046] Siragy, H. M. , R. A. Felder , N. L. Howell , R. L. Chevalier , M. J. Peach , and R. M. Carey . 1989 Evidence that intrarenal dopamine acts as a paracrine substance at the renal tubule. Am. J. Physiol. 257:F469–F477. 2528916 10.1152/ajprenal.1989.257.3.F469

[phy213202-bib-0047] Smith, H. W. , N. Finkelstein , L. Aliminosa , B. Crawford , and M. Graber . 1945 The renal clearances of substituted hippuric acid derivatives and other aromatic acids in dog and man. J. Clin. Invest. 24:388–404. 16695228 10.1172/JCI101618 PMC435470

[phy213202-bib-0048] Tanda, K. , A. Nishi , N. Matsuo , K. Nakanishi , N. Yamasaki , T. Sugimoto , et al. 2009 Abnormal social behavior, hyperactivity, impaired remote spatial memory, and increased D1‐mediated dopaminergic signaling in neuronal nitric oxide synthase knockout mice. Mol. Brain 2:19. 19538708 10.1186/1756-6606-2-19 PMC2711944

[phy213202-bib-0049] Tojo, A. , S. S. Gross , L. Zhang , C. C. Tisher , H. H. Schmidt , C. S. Wilcox , et al. 1994 Immunocytochemical localization of distinct isoforms of nitric oxide synthase in the juxtaglomerular apparatus of normal rat kidney. J. Am. Soc. Nephrol. 4:1438–1447. 7512831 10.1681/ASN.V471438

[phy213202-bib-0050] Tojo, A. , M. Kimoto , and C. S. Wilcox . 2000 Renal expression of constitutive NOS and DDAH: separate effects of salt intake and angiotensin. Kidney Int. 58:2075–2083. 11044228 10.1111/j.1523-1755.2000.00380.x

[phy213202-bib-0051] Verdon, C. P. , B. A. Burton , and R. L. Prior . 1995 Sample pretreatment with nitrate reductase and glucose‐6‐phosphate dehydrogenase quantitatively reduces nitrate while avoiding interference by NADP+ when the Griess reaction is used to assay for nitrite. Anal. Biochem. 224:502–508. 7733451 10.1006/abio.1995.1079

[phy213202-bib-0052] Wang, Z. Q. , H. M. Siragy , R. A. Felder , and R. M. Carey . 1997 Intrarenal dopamine production and distribution in the rat. Physiological control of sodium excretion. Hypertension 29:228–234. 9039107 10.1161/01.hyp.29.1.228

[phy213202-bib-0053] Wang, X. , Y. Luo , C. S. Escano , Z. Yang , L. Asico , H. Li , et al. 2010 Upregulation of renal sodium transporters in D5 dopamine receptor‐deficient mice. Hypertension 55:1431–1437. 20404220 10.1161/HYPERTENSIONAHA.109.148643 PMC2876328

[phy213202-bib-0054] Young, M. K. Jr , and L. G. Raisz . 1952 An anthrone procedure for determination of inulin in biological fluids. Proc. Soc. Exp. Biol. Med. 80:771–774. 12983409 10.3181/00379727-80-19758

[phy213202-bib-0055] Yuste, J. E. , M. B. Echeverry , F. Ros‐Bernal , A. Gomez , C. M. Ros , C. M. Campuzano , et al. 2012 7‐Nitroindazole down‐regulates dopamine/DARPP‐32 signaling in neostriatal neurons in a rat model of Parkinson's disease. Neuropharmacology 63:1258–1267. 22877786 10.1016/j.neuropharm.2012.07.031

[phy213202-bib-0056] Zeng, C. , H. Sanada , H. Watanabe , G. M. Eisner , R. A. Felder , and P. A. Jose . 2004 Functional genomics of the dopaminergic system in hypertension. Physiol. Genomics 19:233–246. 15548830 10.1152/physiolgenomics.00127.2004

[phy213202-bib-0057] Zhang, M. Z. , B. Yao , J. A. McKanna , and R. C. Harris . 2005 Cross talk between the intrarenal dopaminergic and cyclooxygenase‐2 systems. Am. J. Physiol. Renal. Physiol. 288:F840–F845. 15613619 10.1152/ajprenal.00240.2004

